# Efferocytosis signatures as prognostic markers for revealing immune landscape and predicting immunotherapy response in hepatocellular carcinoma

**DOI:** 10.3389/fphar.2023.1218244

**Published:** 2023-06-13

**Authors:** Ke Xu, Yu Liu, Huiyan Luo, Tengfei Wang

**Affiliations:** ^1^ Department of Oncology, Chongqing General Hospital, Chongqing, China; ^2^ Department of Equipment, Bishan Hospital of Chongqing, Chongqing, China

**Keywords:** HCC, efferocytosis, immune, therapy, biomarker

## Abstract

**Background:** Hepatocellular carcinoma (HCC) is a highly lethal liver cancer with late diagnosis; therefore, the identification of new early biomarkers could help reduce mortality. Efferocytosis, a process in which one cell engulfs another cell, including macrophages, dendritic cells, NK cells, etc., plays a complex role in tumorigenesis, sometimes promoting and sometimes inhibiting tumor development. However, the role of efferocytosis-related genes (ERGs) in HCC progression has been poorly studied, and their regulatory effects in HCC immunotherapy and drug targeting have not been reported.

**Methods:** We downloaded efferocytosis-related genes from the Genecards database and screened for ERGs that showed significant expression changes between HCC and normal tissues and were associated with HCC prognosis. Machine learning algorithms were used to study prognostic gene features. CIBERSORT and pRRophetic R packages were used to evaluate the immune environment of HCC subtypes and predict treatment response. CCK-8 experiments conducted on HCC cells were used to assess the reliability of drug sensitivity prediction.

**Results:** We constructed a prognostic prediction model composed of six genes, and the ROC curve showed good predictive accuracy of the risk model. In addition, two ERG-related subgroups in HCC showed significant differences in tumor immune landscape, immune response, and prognostic stratification. The CCK-8 experiment conducted on HCC cells confirmed the reliability of drug sensitivity prediction.

**Conclusion:** Our study emphasizes the importance of efferocytosis in HCC progression. The risk model based on efferocytosis-related genes developed in our study provides a novel precision medicine approach for HCC patients, allowing clinicians to customize treatment plans based on unique patient characteristics. The results of our investigation carry noteworthy implications for the development of individualized treatment approaches involving immunotherapy and chemotherapy, thereby potentially facilitating the realization of personalized and more efficacious therapeutic interventions for HCC.

## 1 Introduction

HCC is the most common type of liver cancer and accounts for a significant proportion of cancer-related deaths worldwide (Yang et al., 2019a). Despite advances in medical treatment, the overall survival (OS) of HCC patients remains suboptimal, and the precise molecular mechanisms underlying HCC prognosis are poorly understood. Currently, HCC prognostic models rely on clinical indicators such as grading and TNM staging, which may have limited accuracy (Icard et al., 2021; Zhai et al., 2022; Wang et al., 2023a; Conche et al., 2023). Therefore, it is imperative to identify novel and effective prognostic biomarkers for HCC, which may help to determine specific therapeutic targets. Molecular immune-targeted therapy represents a promising avenue for future HCC treatment.

“Efferocytosis” refers to the process by which one cell engulfs another cell, usually referring to macrophages engulfing apoptotic cells (Zhou et al., 2020a; Wang et al., 2023b). In addition, there are other cells such as neutrophils, which release DNA fiber networks during the inflammatory process and can engulf these DNA fiber networks and cell debris on DNA (Bukong et al., 2018; Lee et al., 2022); natural killer cells (NK cells), which are usually part of the immune system and can kill infected cells or cancer cells, and can also clear dead cells through efferocytosis (Jensen et al., 2020); malignant tumor cells, some of which can express efferocytosis-related receptors and ligands, enabling them to engulf surrounding apoptotic cells and evade the immune system’s attack (Brightwell et al., 2016; Zhang et al., 2022); dendritic cells and some other immune cells also play a role in efferocytosis (Maschalidi et al., 2022; Nino-Castano et al., 2022). The function of efferocytosis in tumors is complex and can sometimes promote tumor development and other times inhibit it (Banerjee et al., 2021; Tajbakhsh et al., 2021; Lin et al., 2022). In the early stages of tumor development, efferocytosis can promote tumor growth by reducing immune system attacks and promoting the growth of tumor cells by clearing apoptotic cells around them. In addition, tumor cells can further promote tumor development by expressing efferocytosis-related receptors and ligands to evade the immune system’s attack. However, in the late stages of tumor development, efferocytosis can inhibit tumor growth by promoting the immune system’s attack on the tumor.

After engulfing apoptotic cells, antigens in the cells can be presented to T cells by macrophages, thereby activating T cells to attack the tumor (Lu et al., 2022; Zhou et al., 2023). Macrophages play a significant role in the progression of HCC. Recent investigations have revealed significant findings regarding the impact of tumor-derived alpha-fetoprotein (tAFP) on macrophage polarization and its influence on HCC cells. Specifically, tAFP has been shown to promote the differentiation of M0 macrophages into M2 macrophages, while concurrently suppressing the efferocytosis of M1 macrophages towards HCC cells (Zhang et al., 2023a). Polarization of M1 macrophages contributes to the protection against HCC, while M2 macrophages emerges as a prominent factor driving HCC development (Liu et al., 2022a). In view of this, it is necessary to study the role of efferocytosis in the progression of HCC. In addition, efferocytosis can also promote anti-inflammatory responses, thereby inhibiting tumor development (Zhou et al., 2020b). Therefore, the role of efferocytosis in tumors is different from its role in normal physiological conditions and needs to be analyzed according to specific circumstances. In the treatment of tumors, efferocytosis can be used as an important target for intervention to achieve treatment goals (Lahey et al., 2022; Mehrotra and Ravichandran, 2022). However, the role of efferocytosis-related genes in the progression and prognosis of HCC remains poorly understood.

We have developed a risk model based on six efferocytosis-related genes and identified two ERG-associated subtypes that exhibit significant differences in tumor immune landscape and prognostic stratification, highlighting the importance of efferocytosis status in HCC. Importantly, our study reveals patterns of immune therapy and chemotherapy response, and *in vitro* validation confirms the predictive ability of the prognostic model for drug response. These findings underscore the significance of efferocytosis in HCC and suggest potential therapeutic strategies for patients with different efferocytosis statuses. This study may provide a basis for future research on the mechanisms underlying HCC progression and treatment response, as well as inform clinical decision-making in HCC management.

## 2 Materials and methods

### 2.1 Acquisition of TCGA-LIHC data

The Cancer Genome Atlas (TCGA) (https://portal.gdc.cancer.gov/) has aggregated and scrutinized genomic, transcriptomic, epigenomic, and proteomic data obtained from thousands of individuals afflicted with various forms of cancer, culminating in an extensive data repository exceeding 2.5 petabytes. This compendium of knowledge has unveiled potential hereditary drivers of cancer, identified plausible pharmacological targets, and catalyzed the development of customized cancer therapeutics (Wang et al., 2016). We obtained the TCGA-LIHC cohort, comprising transcriptome data of 374 HCC tumor patients and 50 normal liver tissue samples, from TCGA. Additionally, clinical data of 374 HCC tumor patients were downloaded. After rigorous selection, we retained clinical data for a total of 370 HCC patients with comprehensive clinical information.

### 2.2 ERGs from genecards portal

GeneCards is a portal website and database that furnishes a wealth of information on more than 155,000 human genes, encompassing details on gene expression, function, protein domains, and interactions (Safran et al., 2021). Given its comprehensiveness and timeliness, GeneCards represents a valuable resource for investigating the intricacies of human genes and their implications for disease (Sun et al., 2023; Zhong et al., 2023). We employed the following approach to obtain the efferocytosis-related genes. Firstly, we utilized highly relevant keywords and gene descriptions provided in Genecards, such as “efferocytosis,” “phagocytosis of apoptotic cells,” and “clearance of dying cells.” Subsequently, we reviewed the literature to carefully screen and manually confirm these keywords and descriptions to ensure that the final selected genes are indeed closely related to the efferocytosis process. Finally, we obtained a total of 111 genes related to efferocytosis (ERGs) from the GeneCards database.

### 2.3 Prognostic ERGs signature identification

Through the use of univariate Cox regression analysis, we identified a set of 13 genes that displayed a significant correlation with the survival rates of patients with HCC. Then, optimal lambda (λ) was determined to be the ideal value by 10-fold cross validation when performing the LASSO Cox regression analysis to screen the core ERGs that were strongly linked with HCC patients’ prognosis (Chi et al., 2022a; Wang et al., 2022). Using the “glmnet” R package, 6 core genes were subsequently utilized to create a risk signature (Engebretsen, 2019). The risk score was calculated by integrating the expression profile of ERGs with the paired multivariate Cox regression values (β) (Ni et al., 2022; Xu et al., 2022; Zhao et al., 2023a). Based on their respective gene expression profiles, we computed a risk score for each patient in the cohort as follows: Risk score = e^(Exp.GAPDH*0.1481 + Exp.ADAM9*0.1581 + Exp.SIRT6*0.1247 + Exp.LGALS3*0.0666 - Exp.CD5L*0.0144 - Exp.IL33*0.0985).

### 2.4 Evaluating infiltration of immune cells

We employed the CIBERSORT and ssGSEA R scripts to assess the levels of infiltrating immune cells (Newman et al., 2015; Chi et al., 2023a). The CIBERSORT algorithm was used to calculate the immune cell type scores for individual samples, and then the corresponding scores for each sample were calculated based on the estimated immune cell type scores (Chi et al., 2022b). In addition, spearman correlation analysis was used to investigate the relationship between immune cell and risk scores. Using the immune cell profiles of HCC patients, we used the ssGSEA method to distinguish individuals classified as different risks (Zhao et al., 2023b).

### 2.5 Evaluating the accuracy of chemotherapy response predictions

We employed the “pRRophetic” R software package for evaluating the therapeutic response in patient subgroups classified as high-risk and low-risk, based on the half-maximal inhibitory concentration (IC50) values obtained from each individual with HCC from the Genomics of Drug Sensitivity in Cancer (GDSC) dataset (Geeleher et al., 2014; Chi et al., 2023b). Further, the transcriptional profiles of HCC cell lines were obtained from the CCLE website, and risk scores for different HCC cell lines were calculated using the ERGs risk scoring formula. Based on the computed results, Huh7 was identified as having a high risk score, while HepG2 exhibited a comparatively lower risk score. Then, the sensitivity of HCC cells to the drug was evaluated through implementation of the CCK-8 assay (Zhang et al., 2023b).

### 2.6 KEGG and GO analysis

Two frequently utilized bioinformatics resources for investigating the functional and metabolic pathways of genes and proteins, as well as other biological features, are the KEGG and GO databases. Annotations provided by these tools can facilitate a more comprehensive comprehension of gene and protein function, ultimately leading to enhanced insights into gene expression and metabolic regulation. In this study, we performed enrichment analysis using Gene Set Variation Analysis (GSVA) and utilized the “c2.cp.kegg.v7.4.symbols.gmt” data set derived from the MSigDB database (Hanzelmann et al., 2013; Liu et al., 2023a).

### 2.7 Statistical analysis

All data analyses were conducted using R version 4.1.3. For variables that exhibited a normal distribution, the Student’s t-test was employed, whereas Pearson’s correlation coefficient was used to evaluate the association between variables. The levels of statistical significance were set at *p* < 0.05*, *p* < 0.01**, and *p* < 0.001***, respectively.

## 3 Results

### 3.1 Efferocytosis-based gene signature construction

**FIGURE 1:**
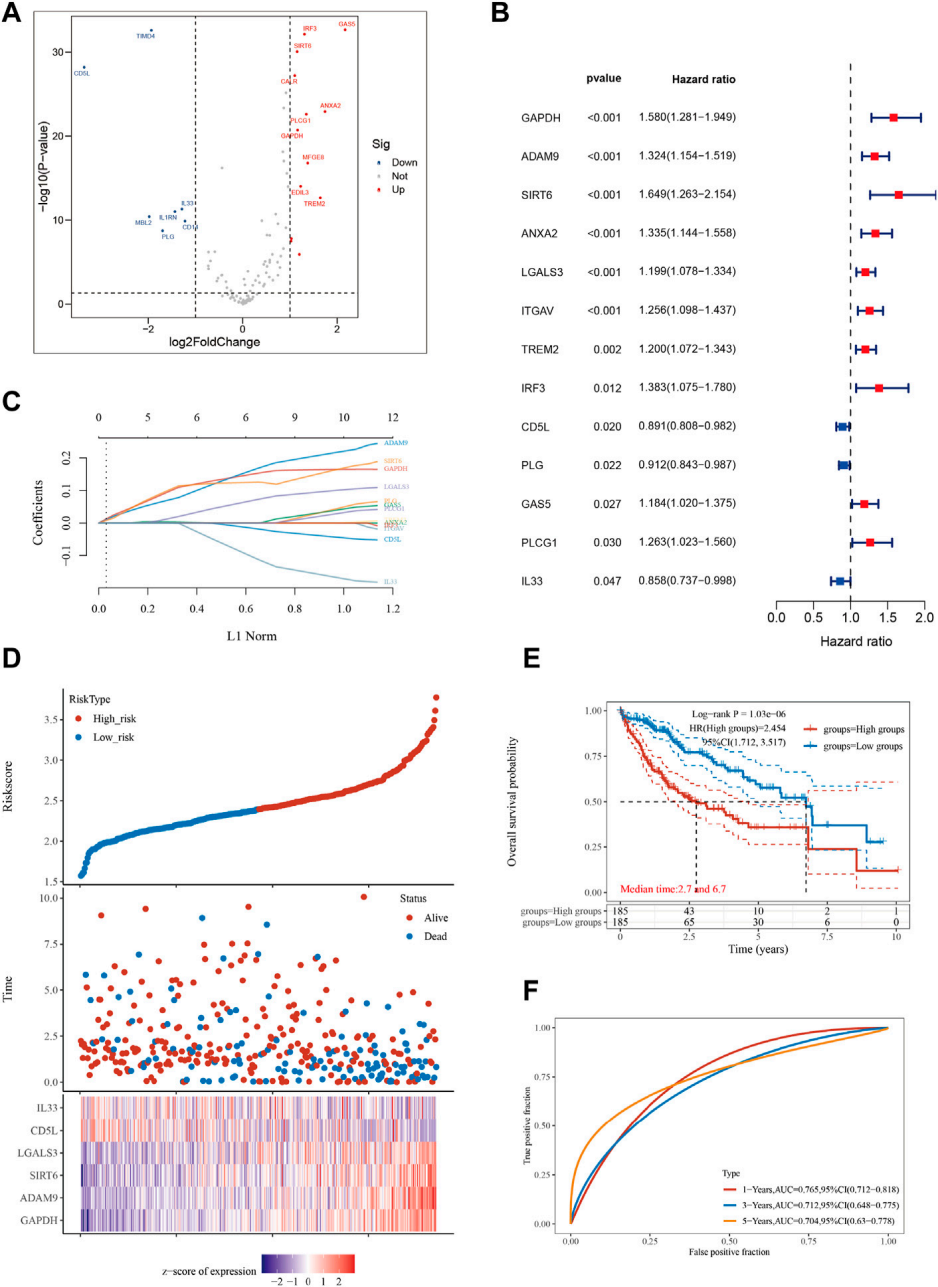
Constructing a prognostic model based on efferocytosis-related genes in HCC. **(A)** Differential gene screening was conducted to identify ERGs associated with hepatocellular carcinoma (HCC). **(B)** 13 genes of prognostic significance, which we refer to as ERGs, were identified from the differential gene screening analysis. These ERGs demonstrated an association with survival in HCC patients. **(C)** Utilizing the Lasso method, a prognostic model was constructed based on the identified ERGs. **(D)** The risk scores, survival status, and expression levels of the top 6-ERGs were plotted to visualize the distribution of prognostic risk. **(E)** Kaplan-Meier (KM) analysis was performed to further investigate the prognostic significance of the 6-ERGs in different HCC subtypes. **(F)** The predictive efficiency of the prognostic model was evaluated using ROC analysis.

We retrieved 111 genes associated with efferocytosis from the Genecards website. The HCC dataset comprising 370 tumor samples and 50 adjacent normal tissue samples was sourced from the TCGA database. We employed the “limma” R package to identify ERGs that were differentially expressed between HCC tumor and adjacent normal samples. This analysis identified 20 ERGs with significant differences (Figure 1A). Next, we utilized the “survival” and “survminer” R packages to investigate the association between ERGs and survival in HCC patients. Thirteen out of the 20 ERGs were significantly linked to survival in HCC patients based on a *p*-value cutoff of less than 0.05 and a km score less than 0.05 (Figure 1B). All ERGs except for CD5L, PLG, and IL33 were found to be poor prognostic factors. To develop an HCC prognostic model, we conducted Lasso analysis using these 13 ERGs (Figure 1C; Supplementary Table S1). The time-dependent ROC curve illustrated the favorable predictive accuracy of the model at 1, 3, and 5 years (Figure 1F). Based on the median riskscore, we divided the 370 HCC patients into high-risk and low-risk subgroups, and the high-risk subgroup displayed a shorter overall survival time than the low-risk subgroup (Figure 1E), with median survival times of 2.7 and 6.7 years, respectively. Furthermore, we generated a heatmap to depict the expression levels of the top 10 ERGs in various riskscore groups (Figure 1D).

### 3.2 ERGs expression variations among subtypes

**FIGURE 2:**
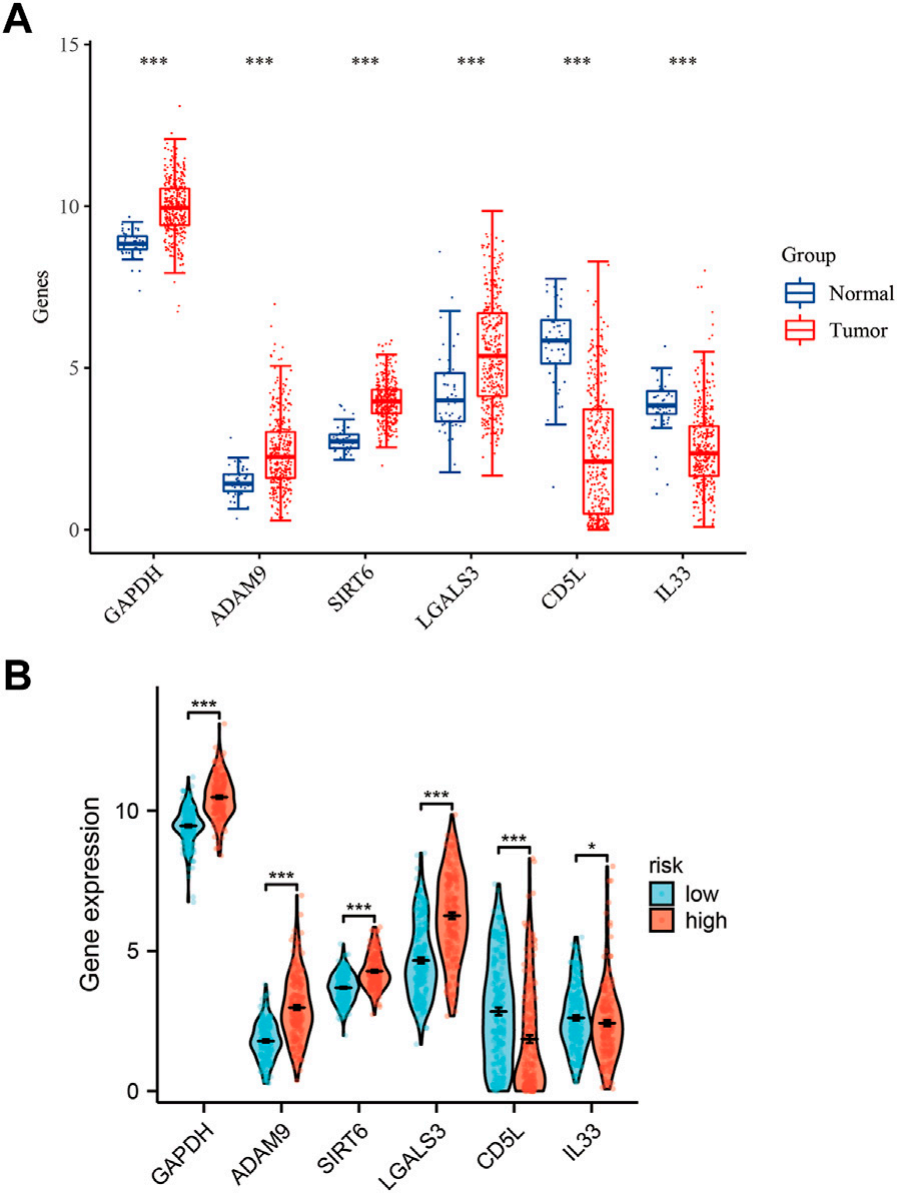
Expression levels of 6-ERGs. **(A)** Expression levels of 6-ERGs in HCC tumor tissues and adjacent tissues. **(B)** Expression levels of 6-ERGs in HCC risk subgroups. (*p < 0.05, **p < 0.01, ***p < 0.001).

Using mRNA expression levels as a metric, we proceeded to assess the expression levels of the six ERGs in both normal and tumor tissues (Figure 2A). Notably, we observed a significantly higher expression of all six ERGs in tumor tissues compared to their adjacent non-tumor counterparts (*p* < 0.001), with GAPDH exhibiting the highest level of expression. To further elucidate the biological significance of these findings, we also examined the expression levels of the six ERGs in high-risk versus low-risk subgroups. Interestingly, we found that the expression trend of the six ERGs in this subgroup mirrored that of Figure 1A (Figure 2B). Moreover, we employed Kaplan-Meier curves to establish the correlation between each key ERG gene and the prognosis of HCC patients, and our analysis revealed that all six ERGs were significantly linked to a poor prognosis (*p* < 0.05).

### 3.3 GO and KEGG enrichment analysis

**FIGURE 3:**
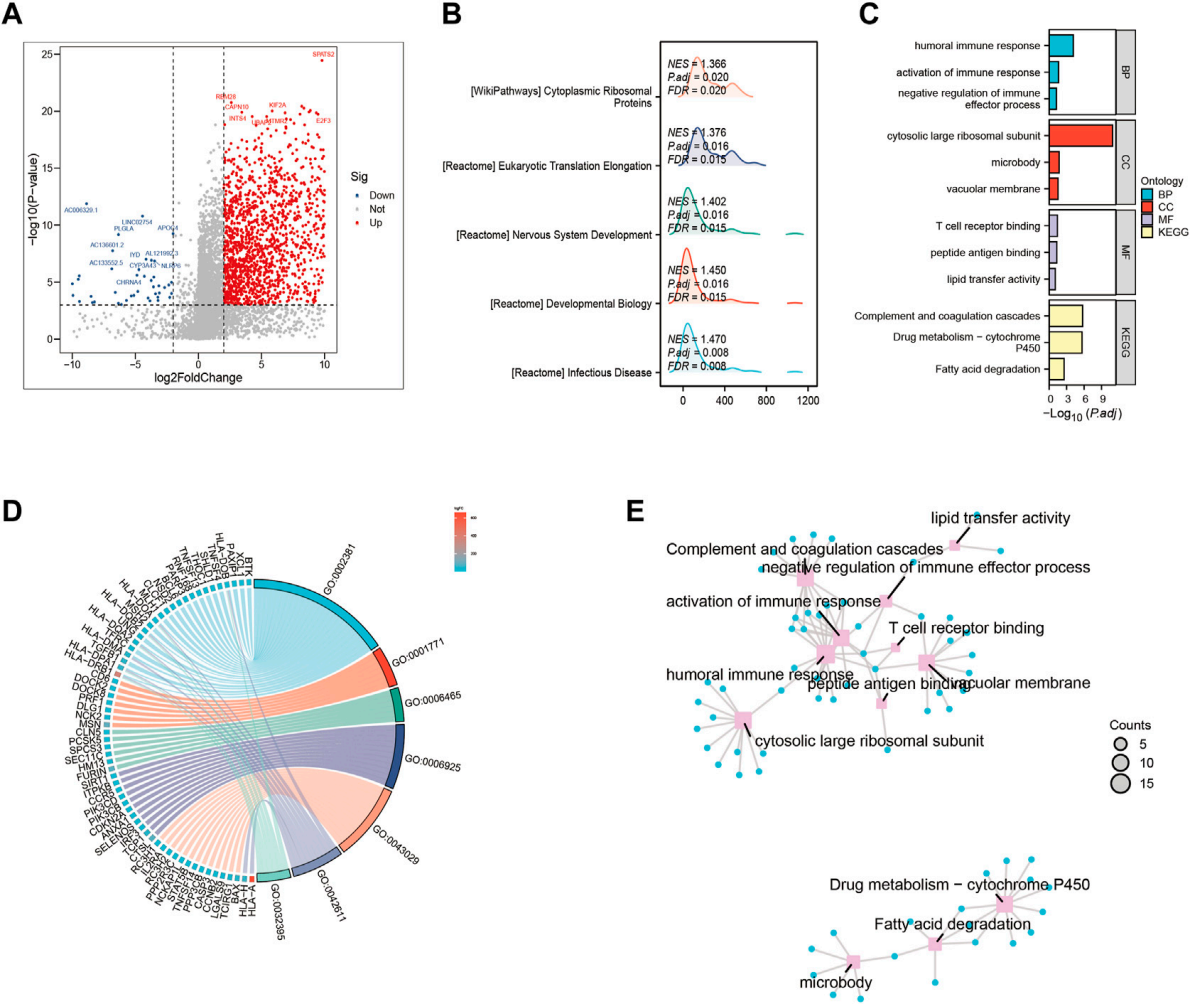
Gene ontology (GO) and Kyoto Encyclopedia of Genes and Genomes (KEGG) analysis **(A)** Volcano map screening for differential genes. **(B)** Mountain map showing the enriched KEGG pathway. **(C–E)** GO enrichment analysis.

We examined the impact of various signaling pathway activations on the growth and progression of tumor cells, as well as their effect on the tumor microenvironment. To identify genes that were differentially expressed between high-risk and low-risk groups, we conducted a comparative analysis of gene expression levels (Figure 3A). In high-risk patients with HCC, several pathways including Cytoplasmic, Ribosomal Protein, Eukaryotic Translation Elongation, Developmental Biology, and lnfectious Disease were significantly enriched (Figure 3B). Additionally, our Gene Ontology enrichment analysis revealed that the humoral immune response process was notably upregulated in the high-risk subgroup (Figure 3C). Furthermore, we investigated the GO pathways that corresponded to the most differentially expressed genes between the high-risk and low-risk subgroups (Figures 3D, E).

### 3.4 Differential immune infiltration levels in HCC patients with diverse risk profiles

**FIGURE 4:**
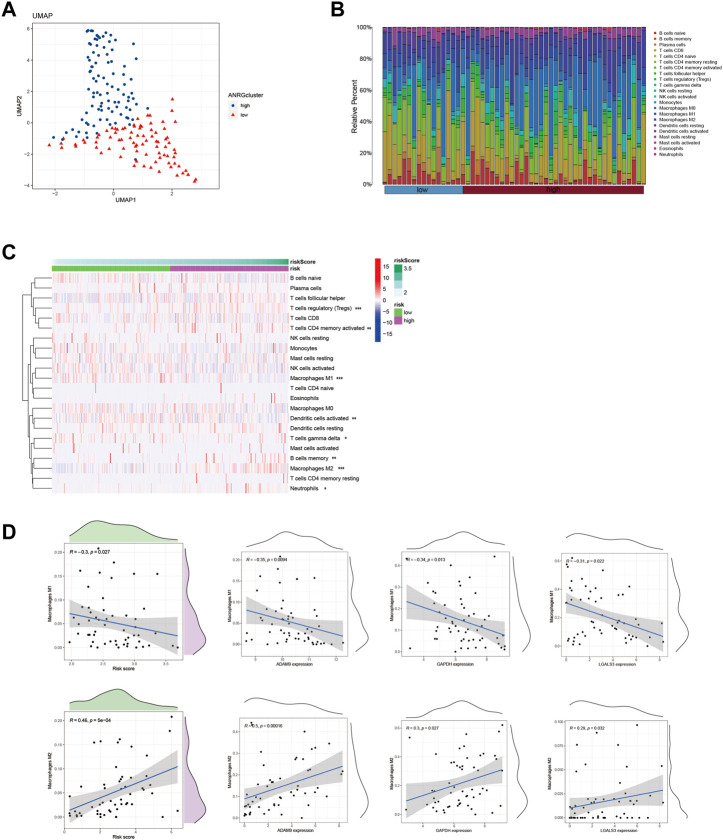
Identify immune landscape of HCC based on efferocytosis-associated signature. **(A)** UMAP demonstrates different immune profiles among HCC subgroups. **(B)** Proportion of immune cells in HCC tissues. **(C)** Differences in immune infiltration between HCC subgroups. **(D)** Correlation between immune cells and 6-ERGs.

Using the Lasso method, we employed dimensionality reduction and clustering on a set of 6-ERGs selected for HCC patients, and our results indicate that these 6-ERGs effectively differentiate between HCC patients of varying risk levels (Figure 4A). Subsequently, we investigated the immune infiltration patterns in HCC patients with distinct prognostic risks (Figures 4B, C). The riskscore values were sorted in ascending order to represent the proportion of each immune cell type (Figure 4B). Remarkably, our analyses revealed significant infiltration of Macrophage M2, activated CD4 memory T cells, and Tregs in HCC patients classified as high-risk, whereas Macrophage M1 was notably decreased in this group (Figure 4C). Additionally, Neutrophils were increased in the high-risk group, suggesting that the HCC patients with high-risk scores may be experiencing an immune-suppressed state, which may be associated with immune checkpoint expression. During our analysis of HCC, we discovered noteworthy distinctions in the expression of both macrophage M1 and macrophage M2 between the high- and low-risk subgroups.

**FIGURE 5:**
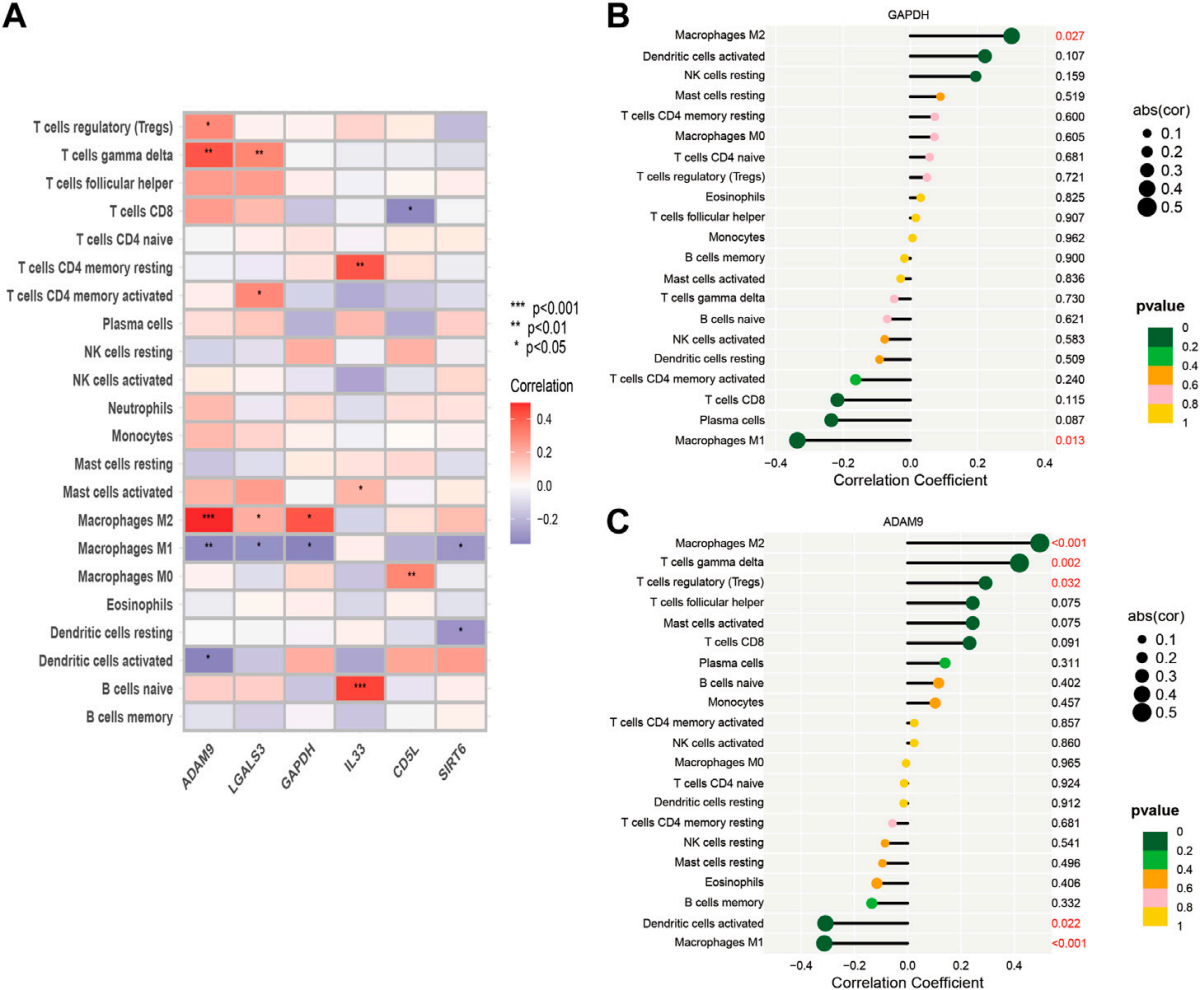
Correlation between immune cells and 6-ERGs. **(A)** Heatmap was used to show the correlation between immune cells and 6-efferocytosis genes (6-ERGs). **(B,C)** Bar plots were used to illustrate the relationship between GAPDH and ADAM9 with immune cell infiltration.

In order to explore this finding further, we sought to investigate the association between macrophage M1 and macrophage M2, and ERGs which have been linked to poor prognosis (Figure 4D). According to the data presented in Figure 4D, the expression levels of ADAM9, GAPDH, and LGALS3 were observed to be positively associated with the abundance of Macrophage M2, while conversely associated with the levels of Macrophage M1. Furthermore, we conducted a more detailed examination of the relationship between the 6-ERGs and immune cells (Figure 5A). Notably, we observed that ADAM9 and GAPDH expression levels exhibited associations with the concentrations of several distinct immune cell types (Figures 5B, C).

### 3.5 Investigating the correlation between ERG expression and immunotherapy efficacy

**FIGURE 6:**
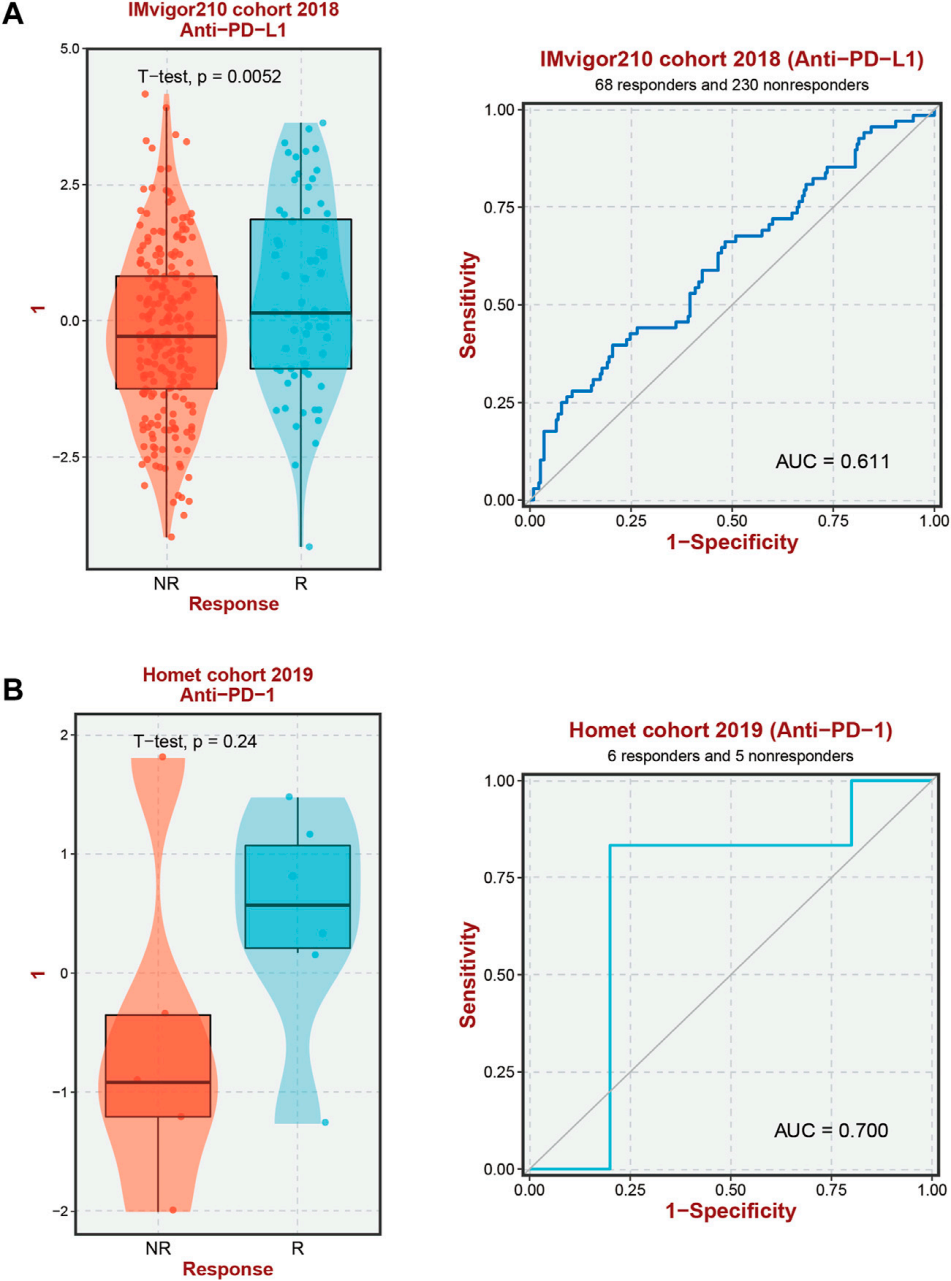
Immunotherapy response prediction. **(A)** Prediction of immune therapy response to anti-PD-L1 treatment in HCC patients based on 6-ERGs. **(B)** Prediction of immune therapy response to anti-PD-1 treatment in HCC patients based on 6-ERGs.

**FIGURE 7:**
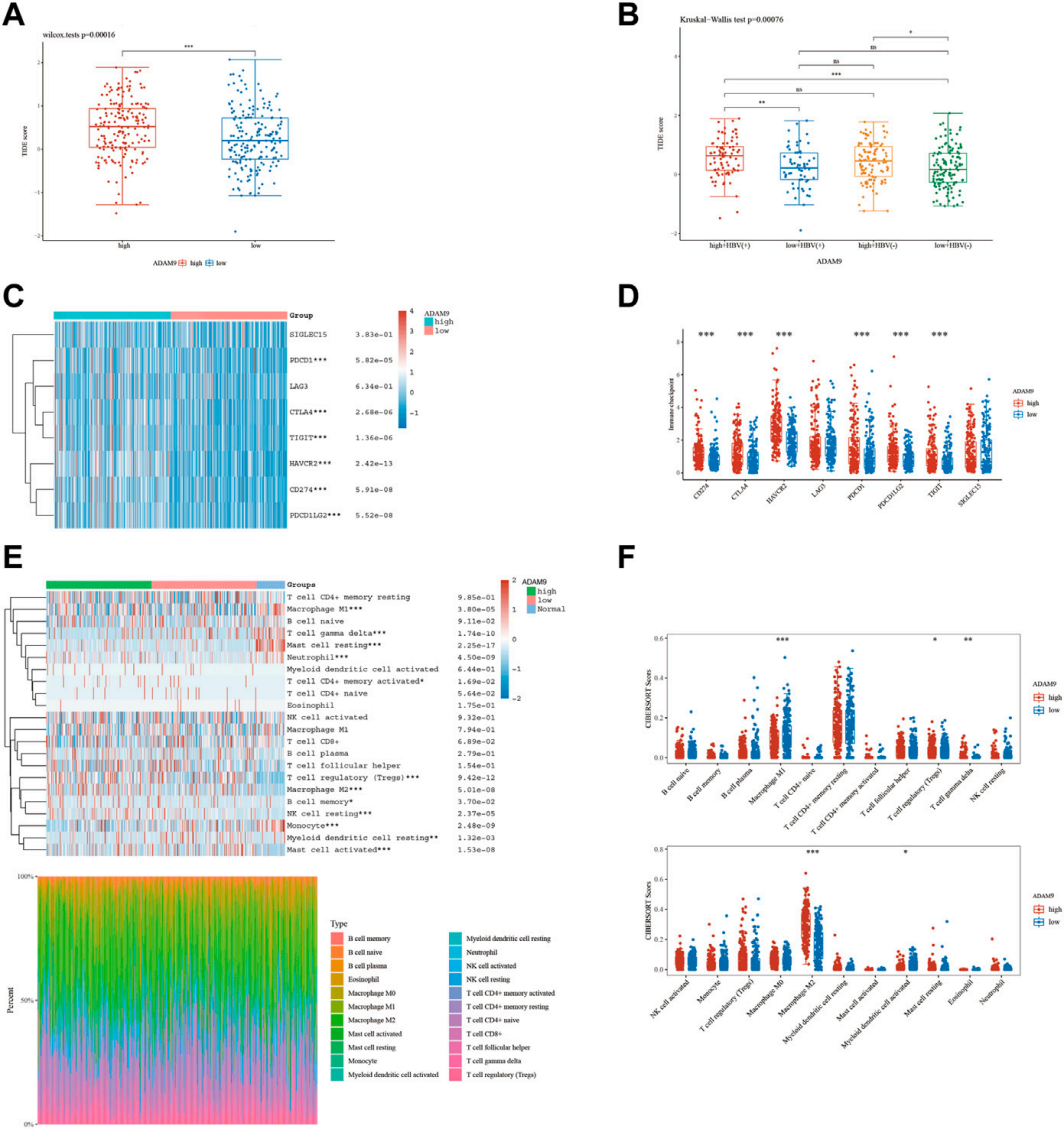
The level of immune checkpoint in HCC subtypes. **(A)** The TIDE score between ADAM9 subgroups. **(B)** HBV infection does not affect the effectiveness of immunotherapy. **(C,D)** There are differences in the expression of immune checkpoint markers between the high-risk and low-risk groups of HCC. **(E,F)** CIBERSORT analysis revealed differences in immune infiltration between the subgroups.

The results of the prior analysis indicate that high-risk and low-risk groups display dissimilar immune microenvironments, characterized by increased infiltration of Tregs, activated CD4 memory T cells, and Macrophage M2 in the high-risk group. These changes create an immunosuppressive microenvironment, which influences the efficacy of immunotherapy differently between the groups. Notably, patients with elevated expression levels of 6-ERGs are more likely to respond to Anti-PD-L1 and Anti-PD-1 therapy (Figure 6). Moreover, 6-ERGs can serve as a predictive tool for the accuracy of immune checkpoint blockade (ICB) in HCC patients (Figures 6A, B). ADAM9 expression gradually increases in cancer tissue and is recognized as a negative prognostic biomarker for prostate cancer patients. Elevated ADAM9 expression is shown to regulate the inflammatory state of the tissue by modulating the efferocytosis of macrophages *in vitro* and *in vivo*. In HCC patient tissues, ADAM9 expression is significantly upregulated (Figure 2A), indicating a higher immune response compared to lower ADAM9 expression subgroups (Figure 7A). To investigate the response of high-risk and low-risk HCC patients to ICB, we used the TIDE algorithm to combine ADAM9 expression levels with HBV infection factors (Figure 7B). We found that high ADAM9 expression predicts a higher immune response score, independent of HBV infection status. Given the effect of ADAM9 on the immune response score, we further explored the expression levels of immune checkpoints in HCC patients with different ERGs riskscores. Surprisingly, we observed that most immune checkpoints, including PDCD1, CTLA4, and PDCD1LG2, are significantly upregulated in the high ADAM9 expression group (Figures 7C, D). Finally, we employed Cibersort to analyze the level of immune cell infiltration in tissue samples categorized into normal, low-risk, and high-risk groups (Figures 7E, F), which revealed significant differences in infiltration levels among the groups.

### 3.6 Prediction and authentication of drug sensitivity

**FIGURE 8:**
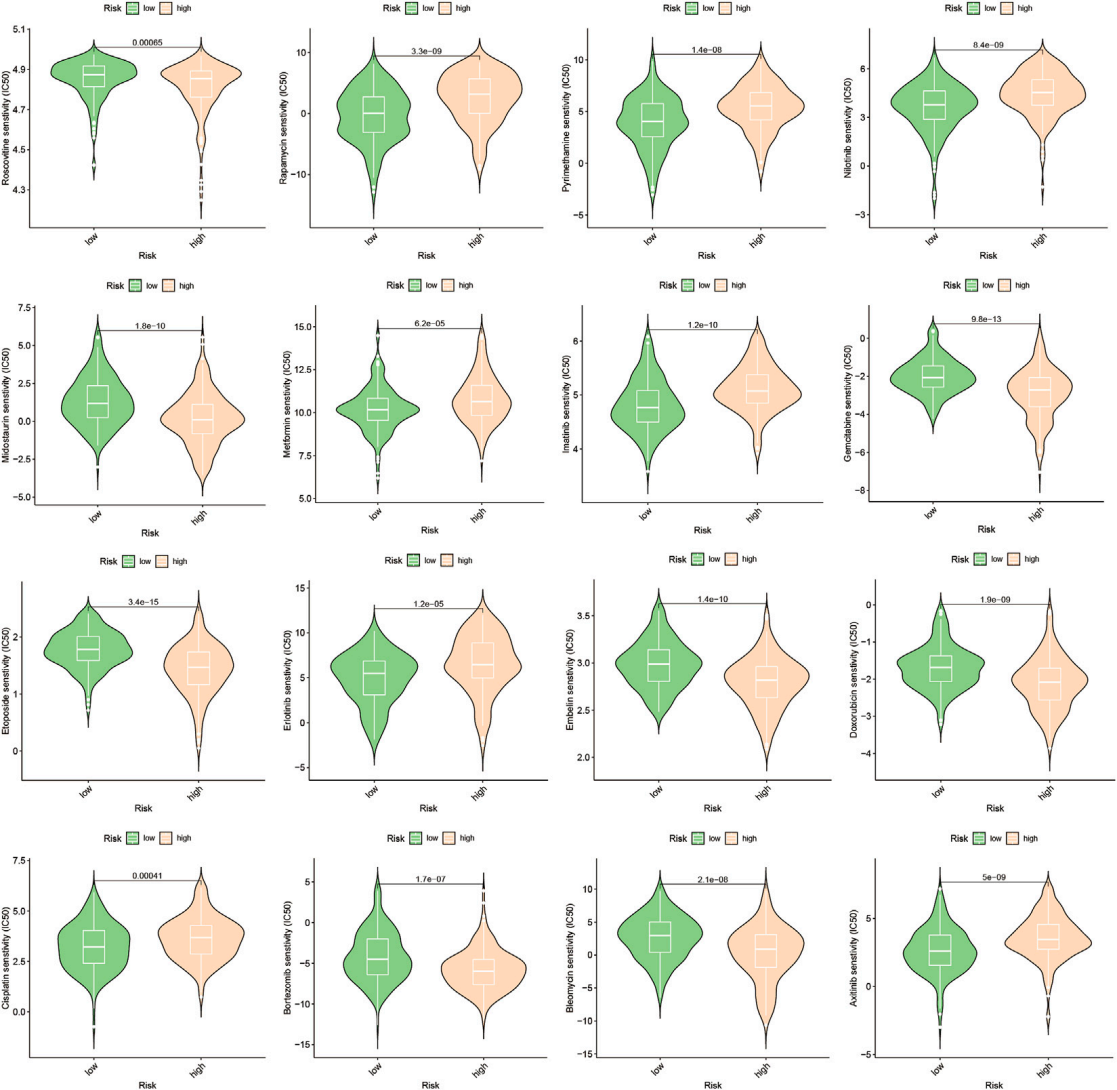
Drug sensitivity prediction.

**FIGURE 9:**
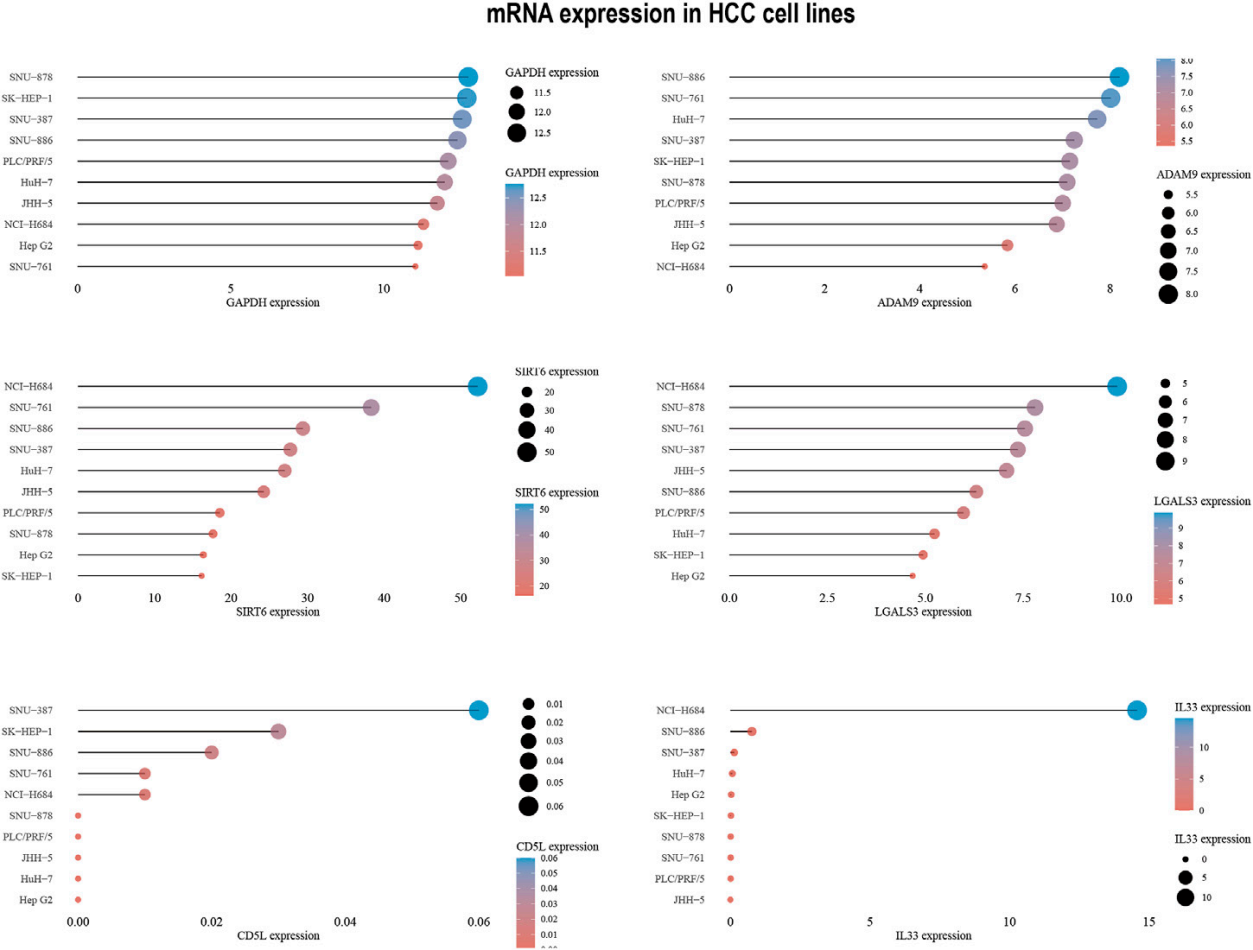
6-ERGs expression levels in HCC cell lines.

**FIGURE 10:**
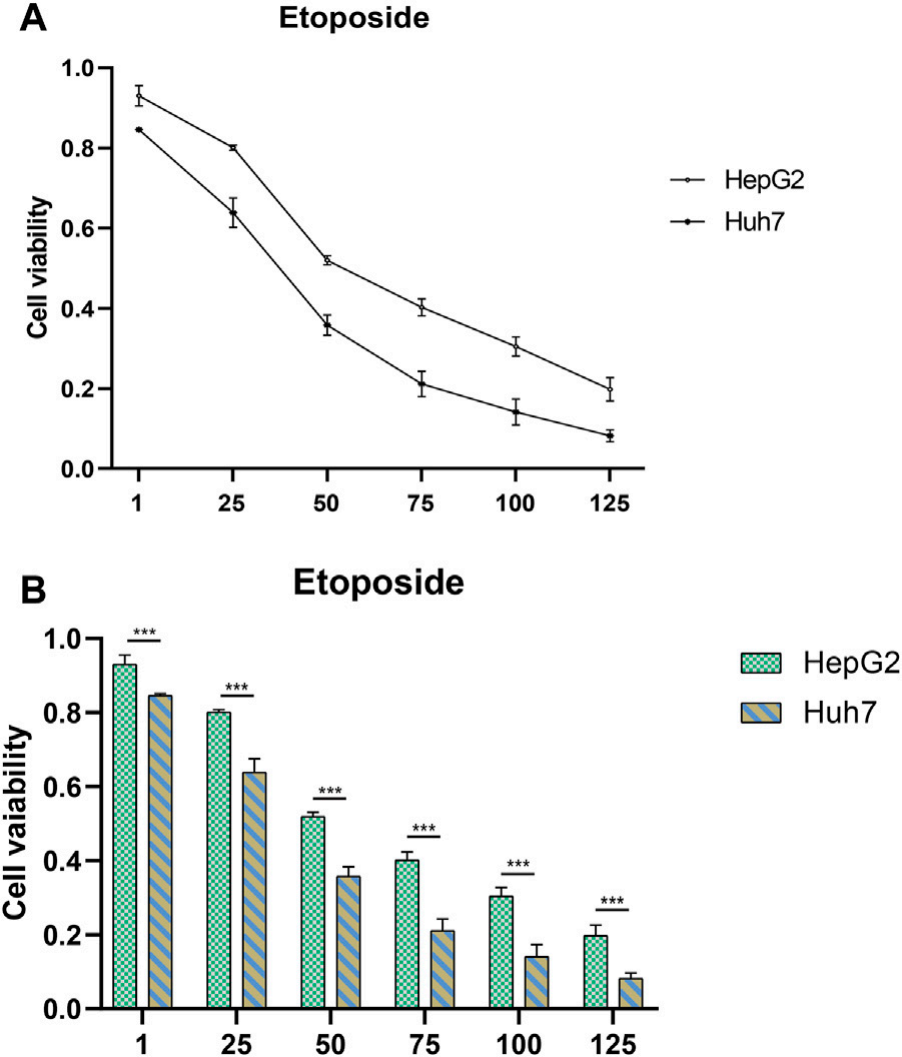
Drug sensitivity testing. **(A,B)** The CCK-8 assay revealed the cytotoxic effects of Etoposide on Huh7 and HepG2 cells at different concentrations.

To develop targeted therapies for patients with HCC, we investigated variations in chemotherapy drug sensitivity between subgroups with high- and low-risk scores. Our analysis compared the IC50 levels of sixteen chemotherapy drugs in the high-risk score and low-risk score subgroups (Figure 8). The results revealed significant differences in IC50 values for some drugs, such as Etoposide, suggesting that patients with high-risk scores may be more responsive to this type of chemotherapy (Supplementary Table S1). To further validate our findings, we assessed riskscores in various HCC cell lines based on gene expression profiles (Figure 9; Supplementary Table S2). For drug sensitivity experiments, we selected Huh7 and HepG2 cell lines to represent the high-risk score and low-risk score subgroups of HCC patients, respectively. Consistent with the drug sensitivity prediction results, our CCK-8 assay data showed that Huh7 cells with a high-risk score were more sensitive to Etoposide than HepG2 cells (Figure 10A), supporting the notion that this chemotherapy drug may be a promising candidate for precision therapy in HCC patients (Figure 10B).

## 4 Discussion

While the rise in the incidence of HCC has shown a decelerating trend in recent times, the morbidity and mortality associated with this disease remain significant (Yang et al., 2019b; Sung et al., 2021). As per current estimates, over 70% of patients who undergo radical resection experience recurrence of the disease within 5 years (Xu et al., 2019; Zhou et al., 2020c). Given these challenges, developing an accurate predictive model for postoperative recurrence and identification of HCC patients with a reduced overall survival is crucial for optimal clinical decision-making and prognostic outcomes.

Efferocytosis is an important process in the immune system, which maintains tissue health by clearing apoptotic cells. It plays a crucial role in both normal physiology and pathological conditions, particularly in tumor progression (Morioka et al., 2019; Myers et al., 2019; Yang et al., 2022). In HCC tissue, tumor cells continuously proliferate, die, and undergo apoptosis, leading to the release of various cytokines and chemicals that trigger inflammatory reactions and immune responses. These reactions lead to the accumulation of macrophages, dendritic cells, and NK cells, which control inflammation and immune responses by clearing apoptotic cells (Yang et al., 2019a; Garcia-Pras et al., 2021; Leone et al., 2021). However, tumor cells can exploit the mechanism of efferocytosis to evade immune system attack (Werfel and Cook, 2018). Studies have shown that tumor cells can express efferocytosis-related receptors and ligands, which attract immune cells to clear apoptotic cells around them and evade immune system attack by interfering with the activation of M1 macrophages and increasing the number of M2 macrophages (Graham et al., 2014; Poon et al., 2014; Soki et al., 2014). Therefore, efferocytosis plays an important role in tumor progression. It has been demonstrated regulation of efferocytosis processes enhances immune cell attack on tumors and increases apoptosis of tumor cells, thereby delaying tumor growth and metastasis (Vaught et al., 2015). Therefore, understanding the relationship between efferocytosis and tumors is of great significance for the development of more effective tumor treatment methods.

Utilizing machine learning to construct a reliable prognostic model based on known efferocytosis-related genes is essential for improving the accuracy of personalized diagnosis and treatment prediction for patients with HCC. Such an approach holds great potential for enhancing the clinical management of HCC patients. We conducted an investigation to identify differential genes from 111 ERGs and subsequently explored their potential prognostic relevance (Figures 1A, B). The identified differential genes were regarded as promising markers with the potential to influence the survival outcomes of HCC patients. Six ERGs, specifically ADAM9, GAPDH, SIRT6, LGALS3, CD5L, and IL33, were selected for the development of an HCC prognostic model (Figure 1C). Our model accurately predicted overall survival of HCC patients at 1, 3, and 5 years, demonstrating its robust predictive ability (Figures 1D–F).

Cell death frequently occurs in solid tumors during malignant progression, and is influenced by the tumor microenvironment (TME), which plays a crucial role in tumor heterogeneity and tumorigenesis (Roy et al., 2018; Gadiyar et al., 2020; Lahey et al., 2022; Li et al., 2023a; Sandri et al., 2023). The nature of cell death and the mechanisms involved in corpse clearance can significantly impact the immune phenotype within the TME (Werfel et al., 2019). The process of efferocytosis, which clears dying cell corpses in the TME, has conventionally been viewed as immunosuppressive (Poon et al., 2014). Our investigation revealed that individuals exhibiting high ERG levels exhibited elevated infiltration of Tregs, activated CD4 memory T cells, Macrophages M2, and neutrophils, in comparison to those with low ERG levels (Figure 4C). Notably, Tregs have been implicated in regulating immune response during the development of HCC, from the early stages to advanced disease. Additionally, Tregs may exert a suppressive influence on liver function, thereby contributing to the emergence of primary liver cancer. The role of M2 macrophages in promoting tumor progression has been widely explored in HCC. The polarization of TAMs towards the M2 macrophage activation state plays a crucial role in anti-inflammatory and pro-tumor activity during tumor progression, which is in stark contrast to the cytocidal and tumoricidal properties of M1 activation states. These fuctions of TAMs partly explain the pronounced enrichment of M2 macrophages in patients with high ERGs, while a divergent trend was observed for M1 macrophages (Figure 4D). The results provide insight into the potential role of macrophage polarization in the prognosis of HCC patients and suggest that targeting ERGs could be a promising therapeutic strategy for the treatment of HCC. However, additional research is needed to elucidate the underlying mechanisms of this relationship and to further evaluate the clinical relevance of these findings.

Immunotherapy has become an essential therapeutic strategy for cancer and has been extensively investigated (Gong et al., 2022; Jin et al., 2022; Zhao et al., 2022; Wang et al., 2023c). This approach involves leveraging the immune system to recognize and eradicate cancerous cells. Numerous types of immune therapies, such as checkpoint inhibitors, adoptive cell transfer, and cancer vaccines, have been developed (Llovet et al., 2022; Peng et al., 2022; Liu et al., 2023b). PD-1 and PD-L1 have been closely associated with macrophages (Liu et al., 2018). Moreover, Abrogation of Efferocytosis leads to diminished immunosuppressive characteristics of macrophages, as evidenced by a reduction in the expression of M2-associated markers such as PD-L1 and PD-L2 (Cruz Cruz et al., 2023). Our study results demonstrate that high ERGs group exhibited elevated PD-1 and PD-L1 expression levels in comparison with the low ERGs group, which could be linked to the macrophage infiltration phenomenon (Figures 7C, D). This observation provides a partial rationale for the superior efficacy of anti-PD-1 and anti-PD-L1 immunotherapy among high ERGs group patients with HCC, emphasizing the important value of the ERGs model in immunotherapy strategies (Figure 6). To further advance the clinical application of this model for predicting anti-PD-1 and PD-L1 response, our subsequent investigations will focus on the evidence from *in vivo* immunotherapy experiments. It is crucial and valuable to assess the accuracy of the ERGs-based model in predicting immunotherapy response by utilizing different risk-scored HCC cell lines or ERGs gene knockout mice. HBV, one of the etiological factors contributing to hepatocellular carcinoma (HCC), exerts a significant impact on the progression of this malignancy. However, in our study, HBV-positive HCC patients did not exhibit significant differences in immune therapy response scores compared to HBV-negative patients (Figure 7B). We postulate that this observation may be closely associated with the viral load of HBV. Despite both groups being HBV-positive HCC patients, variations in viral load could lead to divergent responses to immune therapy. Therefore, overall, HBV-positive HCC patients may demonstrate immune response outcomes comparable to those of HCC patients without HBV infection.

In addition to the interplay between tumor-immune cells, disrupted pathways within tumor cells can also affect the advancement of HCC (Llovet et al., 2018). To gain deeper insights into the differences in pathway enrichment among HCC patients with varying risks, we performed GO and KEGG analyses (Figure 3C). Our findings revealed significant differences in the enrichment of immune response pathways within HCC subgroups classified based on ERGs. These results suggest that ERGs may have an impact on the immune response outcomes of HCC patients (Chang et al., 2013; Fenutria et al., 2014; Braga et al., 2017; Kornberg et al., 2018; Fu et al., 2020; Nie et al., 2020).Our pathway enrichment analysis revealed significant enrichment in cytoplasmic ribosomal proteins, eukaryotic translation elongation, humoral immune response, and cytosolic large ribosomal subunit pathways in HCC patients with high expression of ERGs (Figures 3B–E), which could potentially influence the response of HCC patients to chemotherapy. Drug resistance is also an important factor in persistent tumor progression. Leveraging the six ERGs, we identified sixteen potential clinical drugs that could be tailored to specific subtypes of HCC (Figure 8). We then tested the reliability of our predictions by selecting etoposide as a representative drug. Based on the expression levels of ERGs, HCC cell lines were classified into high and low ERG expression groups, with Huh7 and HepG2 representing the high and low groups, respectively (Figure 9). Our results demonstrated that Huh7 cells exhibited greater sensitivity to etoposide, with a lower IC50, compared to HepG2 cells when treated with various concentrations of the drug (Figure 10A). Furthermore, under identical treatment conditions with the same concentration of etoposide, the drug exhibited greater cytotoxicity to Huh7 cells (Figure 10B). Our findings are in agreement with our initial predictions of drug sensitivity and underscore the reliability of ERGs in predicting chemotherapy response. Based on the drug sensitivity list provided in Supplementary Table S1, the implementation of combination therapeutic strategies involving specific drugs in conjunction with first-line treatments may potentially enhance the anti-tumor therapeutic efficacy for high-risk or low-risk hepatocellular carcinoma patients. It is important to note that prior to implementation, rigorous *in vivo* experiments are required to validate these approaches adequately.

In recent years, the connection between efferocytosis and tumors has garnered notable interest. The expression of glycolytic metabolic genes is known to influence the TME and thus the susceptibility of HCC cells to immunotherapy or chemotherapy. As such, personalized therapeutic approaches should be implemented for cancer patients based on their specific degree of efferocytosis. The classification of tumor samples using gene expression profiling has been well-established as a reliable technique (Werfel et al., 2019; Gong et al., 2022; Zhao et al., 2022). In the present investigation, we classified HCC patients based on the expression levels of efferocytosis-related genes, revealing significant differences in prognostic outcomes and immune infiltration profiles among patients with varying ERG expression levels. Our findings support the use of a six-gene efferocytosis-related model to accurately predict patient prognosis. Furthermore, our cell toxicity assays have confirmed the efficacy of our chemotherapy sensitivity predictions, which could aid clinicians in selecting optimal treatment regimens (Jin et al., 2021; Liu et al., 2022b; Zhong et al., 2022). These results emphasize the capability of our 6-gene model to serve as a reliable prognostic indicator for overall survival in individuals with HCC. Furthermore, our findings suggest that this model could be instrumental in pinpointing novel therapeutic targets for high-risk patient cohorts.

Despite the valuable clinical implications of our investigation regarding prognostic assessment and treatment selection for patients diagnosed with HCC, it is essential to acknowledge the limitations present in our study. Primarily, our research is retrospective in nature, necessitating validation through prospective studies in the future. Due to the unavailability of mRNA expression profile data for HCC patients undergoing immunotherapy, an indirect assessment was conducted to explore the predictive capability of this signature in terms of immunotherapy response. It is important to note that this approach may deviate from the actual circumstances, introducing a potential limitation in the analysis. Consequently, it is imperative to conduct further validation studies that incorporate data obtained from HCC patients undergoing immunotherapy. Besides, the migratory capacity of tumor cells is closely associated with unfavorable prognosis and recurrence (Wu et al., 2021; Li et al., 2023b). However, the relationship between efferocytosis and the migratory potential remains understudied in our investigation. Ultimately, the current study lacks sufficient *in vivo* experiments to enhance the reliability of drug prediction outcomes, thus impacting their potential for further clinical applications. These limitations warrant the need for future investigations aimed at refining and expanding upon these aspects.

## 5 Conclusion

Efferocytosis plays a critical role in both normal physiological processes and pathological conditions, particularly in tumor progression, within the immune system. Despite this, the function of efferocytosis-related genes in HCC progression and prognosis remains largely unexplored. To address this gap, we developed a risk model based on six efferocytosis-related genes and identified two subtypes associated with ERG that demonstrate significant differences in both tumor immune landscape and prognostic stratification. Our results underscore the importance of efferocytosis status in HCC, and have significant implications for predicting patterns of immune therapy and chemotherapy response. Furthermore, *in vitro* validation confirms the model’s predictive ability for drug response, offering important insights into potential therapeutic strategies for patients with different efferocytosis statuses. Overall, our study highlights the crucial role of efferocytosis in HCC and serves as a valuable foundation for further research into HCC progression and treatment response, as well as for guiding clinical decision-making in HCC management.

## Data Availability

The datasets presented in this study can be found in online repositories. The names of the repository/repositories and accession number(s) can be found in the article/Supplementary Material.
